# Effect of a Vibration System on Pain Reduction during Injection of Dental Anesthesia in Children: A Randomized Clinical Trial

**DOI:** 10.1155/2021/8896408

**Published:** 2021-01-30

**Authors:** Muhanad AlHareky, Jehan AlHumaid, Sumit Bedi, Maha El Tantawi, Mazin AlGahtani, Yousef AlYousef

**Affiliations:** ^1^Department of Preventive Dental Sciences, College of Dentistry, Imam AbdulRahman Bin Faisal University, Dammam, Saudi Arabia; ^2^Department of Pediatric Dentistry and Dental Public Health, Faculty of Dentistry, Alexandria University, Alexandria 21521, Egypt

## Abstract

**Background:**

The ‘‘gate control” theory suggests pain can be reduced by simultaneous activation of larger diameter nerve fibers using appropriate coldness, warmth, rubbing, pressure, or vibration. This study investigated the efficacy of a device combining cold and vibration, for needle-related procedural pain in children. *Methodology*. A total of 51 children aged 5–12 years participated in this randomized controlled clinical trial. Half of the children were in the control group and received maxillary buccal infiltration, by injecting 1.8 ml of 2% lidocaine with 1 : 100,000 adrenaline using topical anesthesia 20% benzocaine gel for 15 seconds, while the other half were in the test group and received the same anesthesia using a commercially available external cold and a vibrating device. A face version of Visual Analogue Scale (VAS) was used as a subjective measure to assess the child's pain experience. The parents were requested to evaluate the child's ability to tolerate pain using a behavioral/observational pain scale. Sound, Eyes, and Motor (SEM) scale and Faces, Legs, Activity, Cry, and Consolability (FLACC) scale were used to record the child's pain as perceived by the external evaluator. *T*-test or Mann–Whitney *U*-test was used for scale variables, paired sample *T*-test or Wilcoxon rank *t*-test was used for before and after data, and chi-square was used for categorical variable, based on the results of normality test.

**Results:**

The results showed a statistically significant reduction in pain after the injection for the test group compared with control using VAS scale (mean = 6.68 (1.09) and 8.42 (0.50); *p*=0.001) and FLACC scale (mean = 5.92 (1.05) and 8.16 (0.54); *p*=0.002), but not when using SEM scale (mean 3.22 (0.42) and 4.24 (2.74);*p*=0.08).

**Conclusions:**

Combined external cold and vibrating devices can be an effective alternative in reducing experienced pain and fear in children undergoing infiltration dental anesthesia. This study was registered with clinical trial registry of the United States National Institutes of Health (NIH) at ClinicalTrials.gov (NCT03953001).

## 1. Introduction

Pain is an unpleasant sensory and emotional experience associated with actual or potential tissue damage [1]. One of the most fear-inducing and expected to be painful procedures in pediatric dentistry is the injection of local anesthetic [2]. Effective pain control is the cornerstone for successful behavior guidance in pediatric dental office [3].

Therefore, pain management during dental treatment is of utmost importance as it could significantly alter the physiological signs like hypoxemia, tachycardia [4], psychological symptoms like needle phobia [5, 6], and other emotional consequences. Long-term consequences of needle phobia include the evasion from healthcare settings and noncompliance with needle-related procedures and other health conditions [6].

Up till now, several pharmacological mediations like topical anesthetics, physical methods like cold and acupuncture or devices, and psychological interventions like distraction techniques have been assessed for pain control during needle-related procedures in pediatric patients [7]. Recently, vibrating devices have been used successfully to distract pediatric patients and masking the pain of intramuscular injections and venipuncture [8].

However, the usage of vibration devices to distract patients during dental anesthesia administration has revealed a mixed response in dentistry. Few studies reported that results have not been promising [9–11], whereas others have reported it as a successful technique in alleviation of pain during administration of dental anesthesia [12–15]. Another technique commonly used in medicine to relieve the pain of injection is cooling of the injection site and it has been successfully tested in dentistry also [14].

Recently, a simple and easy-to-use device was developed to prevent pain from needle-related procedures in children. It is a bee-shaped device and consists of a main vibrating body and two removable ice wings [16]. The main vibrating body is power driven by two alkaline (AAA) batteries, which can be started by a switch on the top of the device. The ice wings contain 18 g of ice and are inserted at the back of the body with elastic bands. The device is placed in close proximity to the site of local anesthetic injection and then kept activated throughout the injection period [16].

Current research in dentistry is focused on the usage of cold temperatures in addition to a vibration device [14, 15]. The rationale for using this technique is that, as a psychological component, pain is reliant on the perception and attention of the patient [17, 18].

The aims of this study were to (1) determine whether or not individuals anticipate and report actual pain before and during a buccal infiltration injection and (2) quantify the effect of this device during a possibly painful experience such as buccal infiltration injection.

The null hypotheses were as follows: (1) no pain will be experienced during standard buccal infiltration injection and no difference will be observed between anticipated and actual pain; and (2) application of the device at the site of injection would have no significant consequence on the observation of pain.

## 2. Materials and Methods

### 2.1. Ethical Approval

This study was registered with the clinical trial registry of the United States National Institutes of Health (NIH) at ClinicalTrials.gov (NCT03953001). Ethical approval for the conduct of this study was obtained (IRB-2016-02-100) from the Institutional Review Board of the College of Dentistry, Imam Abdulrahman Bin Faisal University, Dammam. Parents were informed and consent was obtained in writing from them before inclusion of patient in the study.

### 2.2. Study Design

Two parallel arm, randomized controlled clinical trial design was used.

### 2.3. Sample Size Determination

The following assumptions were used to calculate sample size:

1. Expected proportion of reporting pain in the control group = 50%
2. Expected proportion of reporting pain in vibration system group = 20%
3. Alpha error = 5%
4. Study power = 80%

The sample size was calculated using the G-power sample size calculator (University of Kiel, Kiel, Germany). The minimum sample size needed to detect difference between control and vibration system is 45 children. The planned sample size was increased to 50 to make up for losses due to various reasons.

### 2.4. Inclusion Criteria for Participants

1. Children 5–12 years of age.
2. Positive or definitely positive behavior on Frankl scale.
3. Children receiving treatment on the dental chair. Those in need of treatment under general anesthesia were excluded from the study.
4. Free from allergies to topical anesthetic used in the study.
5. Parental consent for child participation in the study.
6. Child is free from any neurological or psychological disorders.

### 2.5. Recruitment

**Figure 1:**
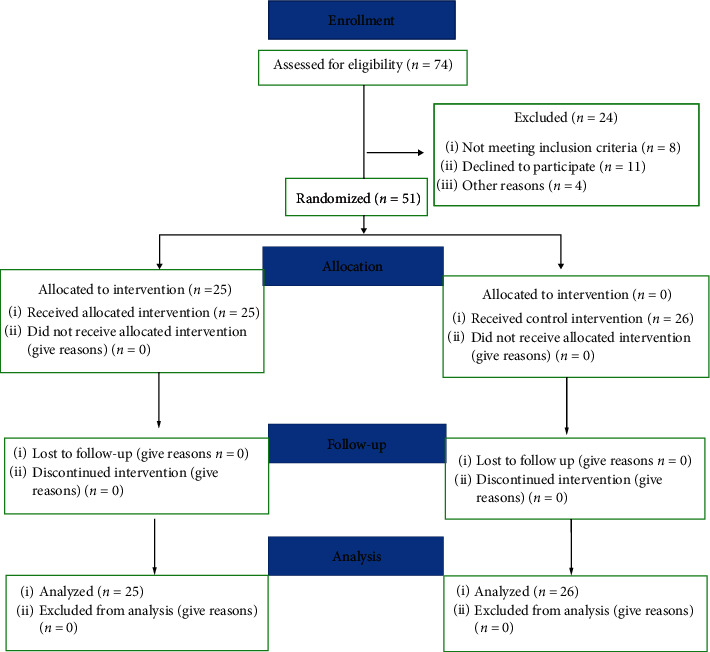
CONSORT diagram showing flow of participants.

Fifty children were recruited from those visiting the clinics of College of Dentistry, Imam Abdulrahman Bin Faisal University, Dammam. Eligibility criteria were applied, and patients included in the study were listed and assigned an identifying number (Figure 1).

### 2.6. Randomization

Patients meeting the inclusion criteria were continuously recruited into the study and were randomly assigned into one of the two study groups, using computer-generated randomization “R 2.11.1 software” (R Foundation for Statistical Computing, Vienna, Austria), to receive the maxillary infiltration injection according to the traditional technique using topical anesthesia only (control group), whereas the Buzzy® device (MMJ Labs, Atlanta, GE, USA) was used along with the topical anesthesia for the test group.

### 2.7. Outcome Assessment

**Figure 2:**
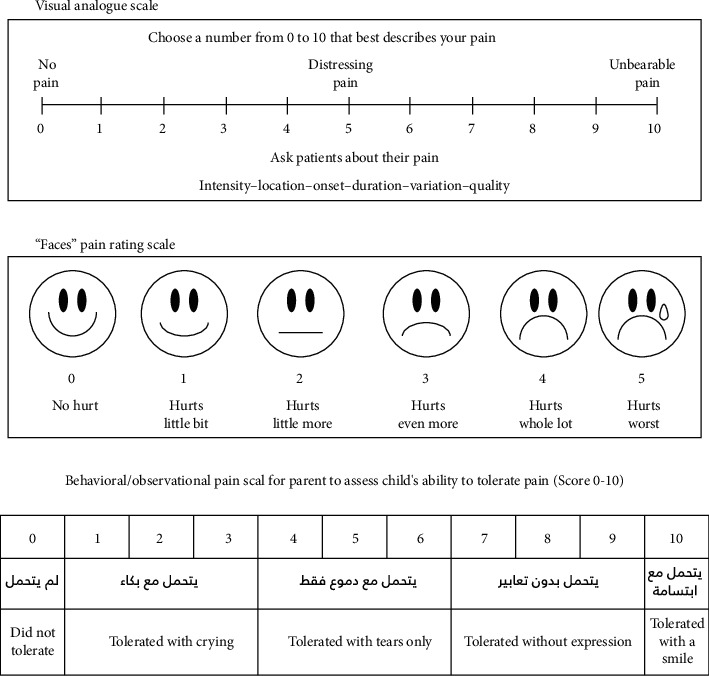
Visual Analogue Scale (VAS) for self-assessment of child's pain perception (score 0–10) and behavioral/observational pain scale for parent to assess the child's ability to tolerate pain (score 0–10).

The anticipated pain and pain during injection were assessed using the self-report pain scale: a face version of Visual Analogue Scale (VAS) [19]. This scale was given to the child to choose twice: the first time was before the injection and the second time was after receiving the injection (Figure 2).

Behavioral/observational pain scale was assessed from two sources: the first source was the parent. Accompanying parent was requested to give a score for the child's ability to tolerate pain during the injection procedure (Figure 2).

**Figure 3:**
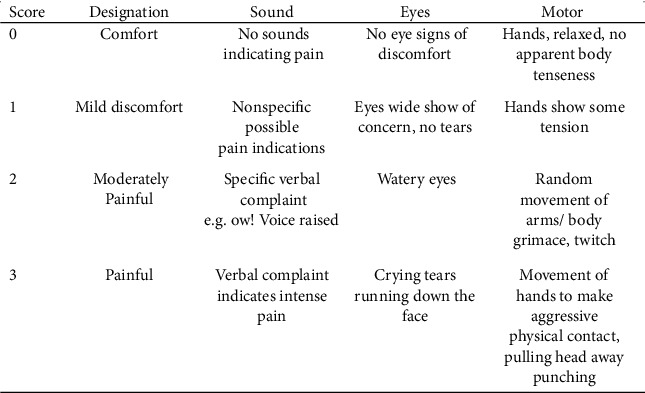
SEM scale for pain assessment from child's behavior (score 0–9).

**Figure 4:**
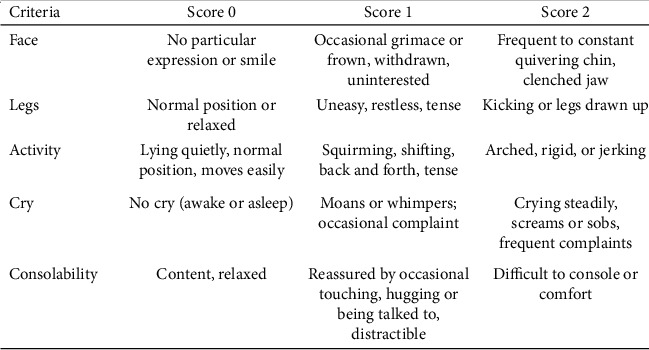
FLACC scale for pain assessment from child behavior (score 0–10).

The second source for evaluating the child behavior during the injection was by using a video filmed for the child before, during, and after the anesthesia using an iPhone *X* camera (Apple Inc.®, USA), and all videos were assessed by an external evaluator, requested to observe the child behavior directly before and after the injection. The evaluator was a recently graduated dentist, trained on the use of the assessment criteria and calibrated with the principal investigator, but it was not possible to blind Buzzy® intervention. The evaluation was made using two validated reliable assessment matrices: Sounds, Eyes, and Motor (SEM) scale (Figure 3) [14] and Faces, Legs, Activity, Cry, and Consolability (FLACC) scale [15] (Figure 4).

## 3. Intervention and Monitoring

### 3.1. Pain with Local Anesthetic Injections

Eligibility criteria were applied, and patients included in the study were listed and assigned a number. Fifty children were recruited from those visiting the clinics of College of Dentistry, Imam Abdulrahman University. Patients were allocated to the control group to receive a maxillary buccal infiltration local anesthetic injection which comprised the administration of 1.8 ml of 2% lidocaine along with 1 : 100,000 adrenaline (Xylocaine, Dentsply, PA, USA) using a 24 mm 30 gauge needle (tgJect, Hammersmith, UK) with a dental anesthetic syringe over the region topically anaesthetized with 20% benzocaine gel for 15 seconds (GINGICaine, Belport County, CA, USA). The test group received the same local anesthesia using the same technique except for using Buzzy® before administration of anesthesia. All procedures were performed by two calibrated board-certified pediatric dentists to eliminate any differences due to the injection technique.

Just before and after the administration of local anesthetic injection, anticipated and actual pain ratings were taken from each child using a 10 cm face version of Visual Analogue Scale (VAS) of pain intensity. Immediately before the first anesthesia attempt in the test group, the trained pediatric dentist attached the ice pack under the device, applied the device against the zygomatic arch before giving maxillary local infiltration anesthesia, and switched on the vibration. The vibration was kept on for the whole length of the injection period. Behavior and pain were assessed by the child himself using VAS and by the parents while they were present in the clinic using a “behavioral/observational pain scale for parent to assess child's ability to tolerate pain” during the procedure. An external evaluator assessed the pain and behavior using the video for the child behavior, before and after the injection. The evaluation was made for children in both groups using Sounds, Eyes, and Motor (SEM) scale and Faces, Legs, Activity, Cry, and Consolability (FLACC) scale.

### 3.2. Analysis

The descriptive statistics were calculated using SPSS version 21 (IBM Corp, New York, USA), in terms of number, frequency, mean, and standard deviations. *T*-test or Mann–Whitney *U*-test was used for scale variables, paired sample *T*-test or Wilcoxon rank *t*-test was used for before and after data, and chi-square was used for categorical variable, based on the results of normality test with significance level (*p* < 0.05).

## 4. Results

A total of 74 patients were assessed for eligibility, and twenty-four patients were excluded: eight did not meet the inclusion criteria, eleven patients declined to participate in the study, and the parents of five patients did not consent for the photography. Fifty-one children were recruited into the study and were randomly assigned into one of the two study groups. 48% of the children in the test group were males compared with 38.5% in control group (*p*=0.49). The mean (SD) age in test group was 8.2 (1.4), and in control group, it was 7.8 (1.6, *p*=0.4). There were no differences between the two groups in terms of the mother's education (*p*=0.25) or father's education (*p*=0.59) (Table 1).

Children in both groups had a positive experience, were comfortable with the dentist and anesthesia (all scores above average, 5/10), and had moderate pain (scores ranging from 4.80−5.52 out of 10). There were no statistically significant differences between the two groups regarding how parents perceived the last dental visit to be positive (*p*=0.13), children's comfort with dentist (*p*=0.84) and anesthesia (*p*=0.34), or pain severity (*p*=0.50) (Table 2).

Table 3 shows the difference between the study groups in the study outcomes. Before injection, children in the test group expressed almost a similar pain to those in the control group on the VAS scale (*p*=0.51), and the evaluator assessed that they had a similar pain experience using the FLACC scale (*p*=0.74) and SEM scale (*p*=0.30). After injection, children in the test group expressed a significantly lesser pain than those in the control group on the VAS scale (mean = 6.68 (1.09) and 8.42 (0.50); *p*=0.001). There was no difference between the two groups in parents' rating of child's tolerance of injection (mean = 7.83 and 7.00; *p*=0.27). The evaluator rated children in both groups after injection using the FLACC scale expressing less pain in the test group compared with the control group (mean = 5.92 (1.05) and 8.16 (0.54); *p*=0.002), but using SEM scale, there was no difference between the groups (mean = 3.22 and 4.24; *p*=0.08).

The amount of change between the pain perception before and after the injection between the two groups was evaluated by counting the difference of rating after the injection compared with the reaction before the injection. The difference of pain rating was significantly higher in the control group compared with the test group when reported by children using VAS scale (mean 5.08 (2.69) and 7.36(2.03); *p*=0.001), but it was not significant when an external evaluator evaluated the child's reaction using FLACC scale (mean 4.34 (2.58) and 6.08 (3.05); *p*=0.145) and SEM scale (mean 1.00 (2.30) and 0.84 (2.13); *p*=0.621) (Table 3).

## 5. Discussion

Local anesthesia is the backbone of pain control and is essential for pain-free dental practice. However, local anesthetic injection is still one of the most anxiety-inducing procedures, especially in pediatrics [4]. Dental fear and anxiety in children are influenced by multiple factors, and hence, several methods, like topical analgesics, distraction practices, warming the anesthetics, and so on, have been recommended to reduce pain and anxiety caused by the local anesthesia [8, 15, 16].

Distraction is a safe and inexpensive behavior management technique that helps reduce the pain and anxiety by diverting the attention from painful stimuli during anesthesia administration [9–11]. Cold and vibration are quick-acting options for distraction and pain relief [14–18].

According to the gate control theory [20], the pain transmitting from the peripheral nervous system to the central nervous system is moderated by a “gate” in the dorsal horn of the spinal cord. The afferent A-delta fibers carrying acute pain and slower C-fibers are blocked by fast nonnoxious motion nerves (A-beta). Therefore, vibrations applied as a counter stimulant to an anesthetic injection will be perceived by the brain before the pain sensation due to injection [20–23]. Additionally, persistent cold application stimulates the C-fibers and may further block the A-delta pain signal [20–22].

This study aimed to assess the efficacy of a vibration device in reducing pain and anxiety amongst children receiving maxillary local infiltration anesthesia, using a parallel-arm randomized controlled clinical trial design. Our results showed that the experimental device demonstrated a statistically significant effect on reducing self-reported procedural pain, observer-reported reaction to pain and anxiety, and parent-reported reaction to pain and anxiety during dental anesthesia related procedures. These results corroborate with the previous study [13] reporting the success with the combined use of cold and vibration to alleviate discomfort and fear in children undergoing maxillary infiltration dental anesthesia. The experimental vibration device is able to reduce oral pain during dental procedures. Therefore, it could be considered as a valuable help for clinicians together with other new improvements that have been demonstrated to reduce oral discomfort, such as digital impressions [24] or photobiomodulation [25].

Self-report is the gold standard in pain assessment in children because, in comparison with the clinical judgment, pain is a subjective experience [23]. In our study, a modified version of VAS using face pictures was used for pain assessment. Previous studies have proved that there is a strong correlation between this modified version of VAS and Wong-Baker scale, which has also been used successfully for children. However, research has shown that Wong–Baker scale may overestimate the pain because anxious children without pain may be unwilling to select a smiling face on the scale [26]. Moreover, clinical judgments are more complex because of the individual characteristics of each patient [27], family history, and related information for pain assessment [28, 29]. Due to the complex nature of the clinical judgments, they are predisposed to human error. Parents knew the characteristic pain reactions of their child and valued contextual and systemic information. Therefore, the inclusion of parents' observations and judgments may offer an enhanced pain assessment compared with that centered on the patient's experience and clinician's observation [30]. Some studies have reported that the parent and child reported pain ratings match, whereas other studies have reported differences [31–33]. In our study, there was no difference between the two groups in parents' ratings of child's tolerance of injection.

The results of this study have an important clinical application in dental practice and corroborate with previous studies showing the benefits of using vibration devices to prevent discomfort in other painful dental procedures. While this study revealed the success of vibration device to decrease pain during injection, the authors believe that other approaches, like patient education and motivation, should also be involved to further reduce the pain.

One of the significant limitations of our study was the inclusion of only cooperative children as this group comprises the major section of pediatric patients in dental practice. However, the findings of our research cannot be generalized to children displaying disruptive behavior in the dental office.

Another limitation of this study is the buccal infiltration technique, which was chosen because it is not reported to be extremely painful [34, 35] and is easy to administer, and there is a minimum discrepancy in the administration of the injection by the clinician [34]. This, however, means that the results of our research cannot be generalized for more painful palatal injections or nerve blocks.

## 6. Conclusion

The experimental device seems to be an easy, useful, and noninvasive intervention to administer dental anesthesia in children. The authors conclude that external cold and vibration application can significantly reduce the experienced pain during maxillary infiltration anesthesia in children. Further research must focus on assessing the efficacy of this procedure for other intraoral sites and techniques of local dental anesthesia, particularly in young children, and this could be undertaken with a larger sample size and including pain assessment by parents also.

## Figures and Tables

**Table 1:** Sample description.

Factors		Group A (test group)	Group B (control group)	*p* value
Sex	Male: *n* (%)	12 (48.0)	10 (38.5)	0.49
	Female: *n* (%)	13 (52.0)	16 (61.5)	

Age	Mean (SD)	8.16 (1.40)	7.83 (1.65)	0.44

Mother's education	Less than high school: *n* (%)	6 (24)	5 (19.2)	0.25
	High school: *n* (%)	6 (24)	12 (46.2)	
	University ad higher: *n* (%)	13 (52)	9 (34.6)	

Father's education	Less than high school: *n* (%)	11 (44)	14 (53.8)	0.59
	High school: *n* (%)	13 (52)	10 (38.5)	
	University ad higher: *n* (%)	1 (4)	2 (7.6)	

Mann–Whitney *U*-test used for age comparison; chi-square test used for all other comparisons.

**Table 2:** Dental history.

	Group A: mean (SD)	Group B: mean (SD)	*p* value
How positive child's last visit was/10	8.93 (1.44)	7.47 (3.25)	0.13
Child being comfortable with dentist during last visit/10	7.38 (3.19)	7.17 (3.45)	0.84
Child being comfortable with anesthesia in last visit/10	6.16 (3.17)	7.16 (3.25)	0.34
Child's pain severity in last visit/10	4.80 (3.40)	5.52 (3.41)	0.50

Mann–Whitney *U*-test used for all comparisons.

**Table 3:** Effect of anesthetic interventions on the study group.

Study outcomes	Group A (test group): mean (SD)	Group B (control group): mean (SD)	*p* value
VAS for pain severity by child before injection (0–10)	1.60 (2.51)	1.08 (1.97)	0.51
VAS for pain severity by child after injection (0–10)	6.68 (1.09)	8.42 (0.504)	0.001^*∗*^
Difference between before and after injection	5.08 (2.69)	7.36 (2.03)	0.001^*∗*^
Child's tolerance of injection by parent/10	7.83 (1.40)	7.00 (3.40)	0.27
FLACC for pain before injection	1.58 (2.58)	2.08 (3.27)	0.74
FLACC for pain after injection (0–10)	5.92 (1.05)	8.16 (0.544)	0.002^*∗*^
Difference before and after injection	4.33 (2.58)	6.08 (3.05)	0.145
SEM for pain before injection (0–9)	4.22 (2.45)	5.08 (3.23)	0.30
SEM for pain after injection (0–9)	3.22 (0.42)	4.24 (2.74)	0.08
Difference before and after injection	1.00 (2.30)	0.84 (2.13)	0.621

^*∗*^Statistically significant at *p* < 0.05; Mann–Whitney *U-*test; Wilcoxon sign rank *t*-test.

## Data Availability

The data used to support the findings of this study will be available from the corresponding author upon request.
